# Comprehensive identification and systematical characterization of *BRX* gene family and the functional of *GhBRXL5A* in response to salt stress

**DOI:** 10.1186/s12870-024-05220-3

**Published:** 2024-06-11

**Authors:** Shouhong Zhu, Yan Li, Wei Chen, Jinbo Yao, Shengtao Fang, Jingwen Pan, Wenting Wan, Javaria Tabusam, Youjun Lv, Yongshan Zhang

**Affiliations:** 1 National Key Laboratory of Cotton Bio-Breeding and Integrated Utilization, Institute of Cotton Research, Chinese Academy of Agricultural Sciences, Anyang, Henan 455000 China; 2 Anyang Institute of Technology, Anyang, Henan 455000 China

**Keywords:** BRX, Cotton, VIGS, Salt stress, Interaction

## Abstract

**Background:**

BRVIS RADIX (BRX) family is a small gene family with the highly conserved plant-specific BRX domains, which plays important roles in plant development and response to abiotic stress. Although BRX protein has been studied in other plants, the biological function of cotton *BRX*-*like* (*BRXL*) gene family is still elusive.

**Result:**

In this study, a total of 36 *BRXL* genes were identified in four cotton species. Whole genome or segmental duplications played the main role in the expansion of *GhBRXL* gene family during evolutionary process in cotton. These *BRXL* genes were clustered into 2 groups, α and β, in which structural and functional conservation within same groups but divergence among different groups were found. Promoter analysis indicated that *cis*-elements were associated with the phytohormone regulatory networks and the response to abiotic stress. Transcriptomic analysis indicated that *GhBRXL2A/2D* and *GhBRXL5A/5D* were up/down-regulated in response to the different stress. Silencing of *GhBRXL5A* gene via virus-induced gene silencing (VIGS) improved salt tolerance in cotton plants. Furthermore, yeast two hybrid analysis suggested homotypic and heterotypic interactions between GhBRXL1A and GhBRXL5D.

**Conclusions:**

Overall, these results provide useful and valuable information for understanding the evolution of cotton *GhBRXL* genes and their functions in salt stress.

**Supplementary Information:**

The online version contains supplementary material available at 10.1186/s12870-024-05220-3.

## Highlights

1. Based on the biological information and systematic analysis of cotton *BRX* gene family, *GhBRXL2A/2D* and *GhBRXL5A/5D* could be used as the candidate genes to validate adversity stress in cotton. Through VIGS verification, *GhBRXL5A* was a negative regulator of salt stress in cotton.

2. GhBRXL1A and GhBRXL5D have homotypic and heterotypic interactions through yeast two-hybridization assays.

**Supplementary Information:**

The online version contains supplementary material available at 10.1186/s12870-024-05220-3.

## Background

BRVIS RADIX (BRX) family is a small, unique and specific gene family in plants, which plays an important role in regulating plant development and responding to abiotic stress [1–9]. BRX protein contains three highly conserved domain regions, one of which is located at the N-terminal BRX-N domain (IPR027988), involving in the membrane localization of BRXL [1–4]. Two other highly conserved tandem repeat domains, the BRX domains (IPR013591) are at the C-terminal, and are the protein–protein interaction domains that play a key role in BRX activity [2]. The function of *BRX* genes have been reported in several plant species such as *Arabidopsis* [1–4], rice [5], *Brassica rapa* [10], and wheat [11].

A total of five *BRX* genes have been identified and found in model plant *Arabidopsis*, namely AtBRX, AtBRXL1, AtBRXL2, AtBRXL3, and AtBRXL4 respectively [2, 12]. Although these *AtBRX* family genes are highly conserved on the coding sequence level, their functions are largely non-redundant [2, 12]. Previous studies have shown that only *AtBRXL1* rescues the phenotype of *brx* mutants, whereas *AtBRXL2-AtBRXL4* only partially rescues *brx* mutants [2, 4]. Similarly, complementation studies showed that only *BrBRX.1* in *Brassica rapa* could rescue the *brx* root phenotype in *Arabidopsis*, but not *BrBRX.2* and *BrBRX.3* [10]. However, complementation studies with rice *OsBRXL1–3* revealed that all the genes could rescue the *brx* mutant of *Arabidopsis* [12]. This indicates that the *BRXL* genes have undergone more functional diversification in *Arabidopsis*, and that the monocotyledonous *BRXL* genes may have similar functions to *AtBRX*. *AtBRX* gene uniquely controls the extent of cell proliferation and elongation in the root tip growth zone [1]. Further studies have shown that *AtBRX* was involved in a variety of signaling pathways mediated by phytohormones (e.g., auxin, brassinosteroid (BR), abscisic acid (ABA) and cytokinin) associated with root growth and development [3, 13–16]. The mutant *brx-2* is highly sensitive to ABA-mediated root growth inhibition and can be restored by BR treatment, suggesting that there is an interaction between ABA and BR in root growth regulation [13]. The *brx-2* mutant is also insensitive to cytokinin-induced inhibition of lateral root formation, and that this can be restored by embryonic BR treatment, suggesting a signaling exchange between BRX-mediated BR and cytokinin pathways [3]. These results suggest that BRX has an important function in regulating phytohormone responses and biosynthesis. Furthermore, the *BRXL* genes have some functions in abiotic stress responses. Except *OsBRXL6*, the other five *OsBRXL* genes are differentially responsive to major stresses including drought, salt, and cold [5]. The growth of *brx* mutants under salt stress was significantly better than that of wild type both after transfer and germination on pH 4.5 [6]. The regulatory mechanism of these *BRXL* responses to stress needs further analysis in the future.

Furthermore, some *BRXLs* are also involved in growth and development of aerial tissues such as leaf and shoot. The *brx* mutants have shown a curled leaf phenotype with significantly reduced cotyledon and leaf growth, while overexpression of *AtBRX* leads to epinastic leaf growth [4]. Ectopic expression of three *BrBRX* genes (*BrBRX.1*-*BrBRX.3*) in *Arabidopsis* have shown epinastic leaf morphology, with increased leaf number and petiole length, and reduced leaf angle [10], suggested that these *BrBRX* genes may be involved in the leaf-heading trait in *Brassica rapa*. Recent studies have shown that OsBRXL4, which is homologous to AtBRX, could also regulate shoot gravitropism and rice tiller angle through affecting the nuclear localization of LAZY1 [7]. *Arabidopsis* branch angle and gravitropism are controlled through BRXL4-LAZY1 interaction at the plasma membrane [8, 9]. The role of *BRXL* in shoot and leaf growth makes these genes important candidates for future crop improvement.

Cotton is an important cash crop in the world, not only providing the materials for industrial production such as cotton fiber and seed, but also a pioneer crop for improving saline-alkali soil. Currently, the release of genome sequences from different plant species such as cotton provides the information for investigating the *BRX* gene family and their functional characteristics in polyploid genome [17–21]. Therefore, in this study, genome-wide analysis of *BRX* genes was performed in cotton and other model plants. The evolutionary relationships were determined by phylogenetic and systematic analysis between cotton and other plant species. Gene structure, promoters, and expression analysis under different developmental and abiotic stress conditions of *BRXL* gene family were analyzed using bioinformatics techniques. Further, a member of the cotton *BRX* gene family, *GhBRXL5A*, was silenced using VIGS technology to elucidate its potential function in response to salt stress. Finally, the protein–protein interactions were analyzed by yeast two-hybridization. These findings provided basic information toward the functional characterization of *BRXL* genes in cotton.

## Results

### Identification and characterization of the BRXL proteins

Based on previously published information of AtBRX and OsBRXL proteins, a total of 76 BRXL proteins were identified by using BLASTP, Interproscan 5 and SMART software within 11 plant species, including 12 *GhBRXLs* from *Gossypium hirsutum* (ZJU), 12 *GbBRXLs* from *G. barbadense* (ZJU), 6 *GaBRXLs* from *G. arboreum* (CRI), 6 *GrBRXLs* from *G. raimondii* (JGI), 6 *OsBRXLs* from rice, 8 *ZmBRXLs* from *Zea mays*, 5 *SbBRXLs* from *Sorghum bicolor*, 4 *VvBRXLs* from *Vitis vinifera*, 4 *PtBRXLs* from *Populus trichocarpa*, 3 *TcBRXLs* from *Theobroma cacao*, 10 *GmBRXLs* from *Glycine max*, respectively. Especially, the number of *BRXL* genes in allotetraploid cotton (*G. hirsutum* and *G. barbadense*) is twice that of diploid cotton *G. arboreum* and *G. raimondii* [22], respectively, indicating that the cotton *BRXL* genes have undergone expansion during evolution.

Further characteristics of all 81 *BRXL* genes including gene sizes, isoelectric points (PI), molecular weights (MW) and subcellular localization were analyzed. These *BRXL* genes showed CDS lengths ranging from 258 (Glyma.01G024200.1) to 1323 (Zm00001d026237) bp, with encoded BRXL proteins sequence length ranging from 85 (Glyma.01G024200.1) to 440 (Zm00001d026237) amino acids (a.a.), with pIs ranging from 4.82 (OsBRXL6) to 9.51 (OsBRXL2), and with molecular weight ranging from 9.43 (Glyma.01G024200.1) to 47.27 (Zm00001d026237) kDa (Additional file 1: Table S1). Among them, four cotton species of 36 cotton *BRXL* genes were named as *BRX like* (*BRXL*) genes based on their orthologs in *Arabidopsis* and rice, “A” or “D” were appended to the gene names to distinguish their chromosomal position at the At or Dt sub-genome (Additional file 1: Table S1). These cotton *BRXL* genes CDS lengths ranging from 1029 (*GhBRXL1A*, *GhBRXL1D*, *GbBRXL1A*, *GbBRXL1D*, *GaBRXL1* and *GrBRXL1*) to 1218 bp (*GrBRXL4*), with protein sequences length ranging from 342 (GhBRXL1A, GhBRXL1D, GbBRXL1A, GbBRXL1D, GaBRXL1 and GrBRXL1) to 405 (GrBRXL4) amino acids, with pIs ranging from 5.73 (GhBRXL1A, GhBRXL1D, GbBRXL1A, GbBRXL1D, GrBRXL1 and GaBRXL1) to 8.59 (GhBRXL5A, GbBRXL5A and GaBRXL5). Molecular weight was ranging from 38.58 (GhBRXL1D, GbBRXL1A, GbBRXL1D, GrBRXL1 and GaBRXL1) to 45.59 (GrBRXL4) kDa (Additional file 1: Table S1). We found that all BRXL proteins were located in nuclear, which is consistent with the function of BRXLs (Additional file 1: Table S1). These results demonstrated that BRXLs regulated the biological processes in organelles.

### Phylogenetic analysis of *BRXL* genes

**Fig. 1:**
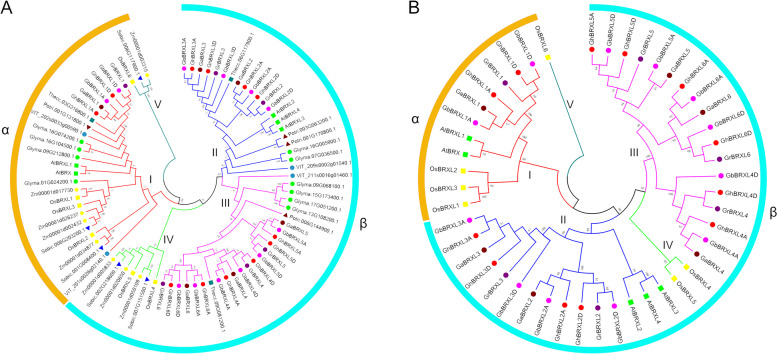
Phylogenetic analysis of BRXL family members. **A** Phylogenetic relationship of the 81 identified *BRXL* genes from 12 plant species. **B** Phylogenetic relationship of the 47 identified *BRXL* genes from four cotton species, rice and *Arabidopsis*. The neighbor joining (NJ) method based phylogenetic trees were generated using MEGA 11.0 software with 1000 bootstrap replicates. Various colors indicate different subgroups of *BRXL* genes

To understand the evolutionary relationship of the *BRXL* gene family in plant species, we constructed the phylogenetic tree using the conserved amino acid sequences of BRXL from 12 species. Based on protein structure and similarity, BRXLs were divided into two groups, α and β (Fig. 1A). The α group was further divided into 2 subgroup I and V. And the β group was divided into 3 subgroups II, III and IV (Fig. 1A). The V subgroup contained the fewest BRXL proteins, only 3 in total. I and III subgroups aggregated the most BRXL proteins, with 25 BRXL proteins in each subgroup. Moreover, except for subgroup I which contained both monocotyledonous and dicotyledonous BRXL proteins, subgroup V and IV only contained monocotyledonous BRXL proteins, and subgroups II and III contained only dicotyledonous BRXL proteins. Further, the phylogenetic tree constructed using only the conserved amino acid sequences of four cotton species and the model plants *Arabidopsis* and rice BRXs were characterized by dividing the phylogenetic tree of all cotton BRXL proteins into five well-defined subgroups (Fig. 1B). Similarly, except for subgroup I which contained both monocotyledonous and dicotyledonous BRXL proteins, subgroup V and IV only contained monocotyledonous BRXL proteins, and subgroups II and III contained only dicotyledonous BRXL proteins. III subgroup aggregated the most BRXL proteins, with 18 BRXL proteins in the subgroup. In subgroups I, II, and III, each BRXL from the two diploid cotton species, *G. arboreum* and *G. raimondii*, respectively, and the two BRXLs from the two allotetraploid cotton species, *G. hirsutum* and *G. barbadense* are in the same branch, suggesting that they are most closely related.

### Chromosomal localization and collinearity analysis of cotton *BRXL* genes

These cotton *BRXLs* were distributed in 28 chromosomes of cotton genome (Additional file 7: Fig. S1). 6 *GrBRXLs* and 6 *GaBRXLs* were distributed in 5 chromosomes of the two diploid cotton *G. arboreum* genome and *G. raimondii* genome, respectively (Additional file 7: Fig. S1A, B). The chromosome Ga-A05 of diploid *G. arboreum* genome and the chromosome Gr-D09 of *G. raimondii* genome had the greatest number of *BRXLs*, with 2 (*GaBRXL2* and *GaBRXL4*, *GrBRXL2* and *GrBRXL4*) in each. And 12 *GhBRXLs* and 12 *GbBRXLs* were distributed in 9 chromosomes of the two *G. hirsutum* and *G. barbadense* allotetraploid cotton genome, respectively (Additional file 7: Fig. S1C, D). Among them, 3 *GhBRXLs* and 3 *GbBRXLs* (*GhBRXL2*, *GhBRXL3* and *GhBRXL4*, *GbBRXL2*, *GbBRXL3* and *GbBRXL4*) were distributed in Gh-A05 and Gb-A05 chromosomes had the greatest number of *BRXLs*.

**Fig. 2:**
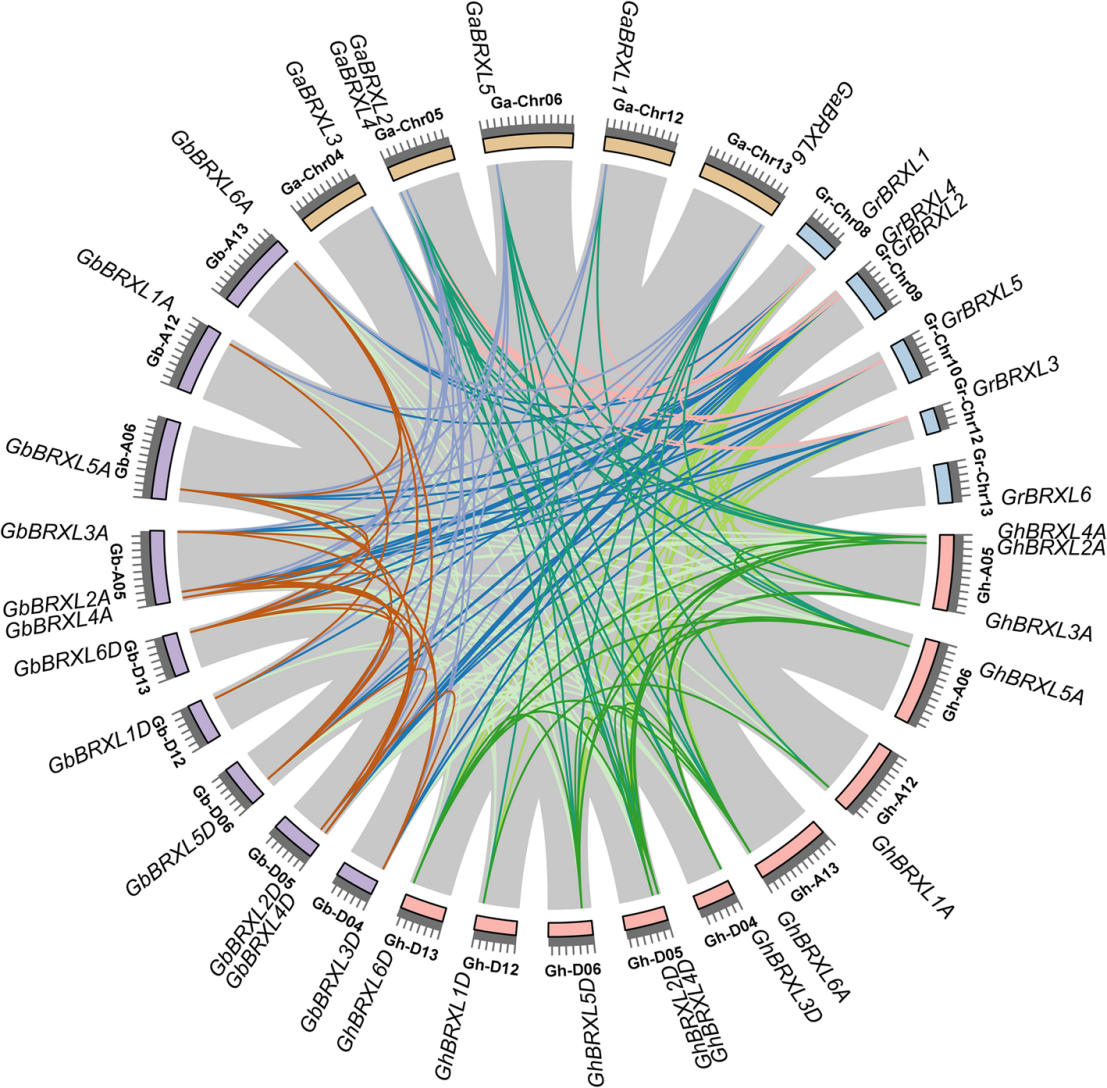
Syntenic relationships among *BRXL* genes of four cotton species. The chromosomes of *G. arboreum*, *G. raimondii*, *G. hirsutum* and *G. barbadense* were shown with orange, blue, flesh and plum red colors, respectively. Each panel in the chromosome length scale of four cotton species represents 10 Mb. WGD/segmental duplication events and inter-chromosomal relationships between *BRXL* genes in four cotton species were investigated with MCScanX and linked by the colored lines, respectively. The gray lines indicated all putative segmental duplication pairs in the cotton genome

Identification of tandem duplication and whole genome duplication (WGD)/segmental duplication were performed in four cotton species by multiple and pairwise alignments of *BRXLs*, to clarify the mechanism of expansion of *BRXL* gene family in cotton. The synteny analysis revealed that a total of 293 orthologous/paralogous gene pairs were identified in cotton species, and most of *BRXLs* were highly conserved among different cotton species (Fig. 2). Except for *GrBRXL6*, which had no orthologous/paralogous gene pairs with other cotton species, orthologous/paralogous gene pairs of *BRXL* genes were found in all cotton species. Among which 42 and 40 pairs were predicted in WGD/segmental duplication to form paralogous gene pairs within the Gh/Gh and Gb/Gb genomes, respectively (Fig. 2; Additional file 2: Table S2). And 44 and 46 pairs were predicted in WGD/segmental duplication to form orthologous gene pairs within the Ga/Gh and Ga/Gb genomes, respectively (Fig. 2; Additional file 2: Table S2). It was predicted that 30 pairs each were involved in WGD/segmental duplication, forming orthologous gene pairs within the Gr/Gh and Gr/Gb genomes (Fig. 2; Additional file 2: Table S2). From these results it presumed that orthologous/paralogous gene pairs were generally raised from WGD/segmental duplication during polyploidization involved in evolution process. Based on collinearity analysis, these results further confirmed that the two allotetraploid cotton species, *G. hirsutum* and *G. barbadense*, are the result of polyploidization between the ancestors of the two diploid cotton species, *G. arboreum* and *G. raimondii* [22].

**Fig. 3:**
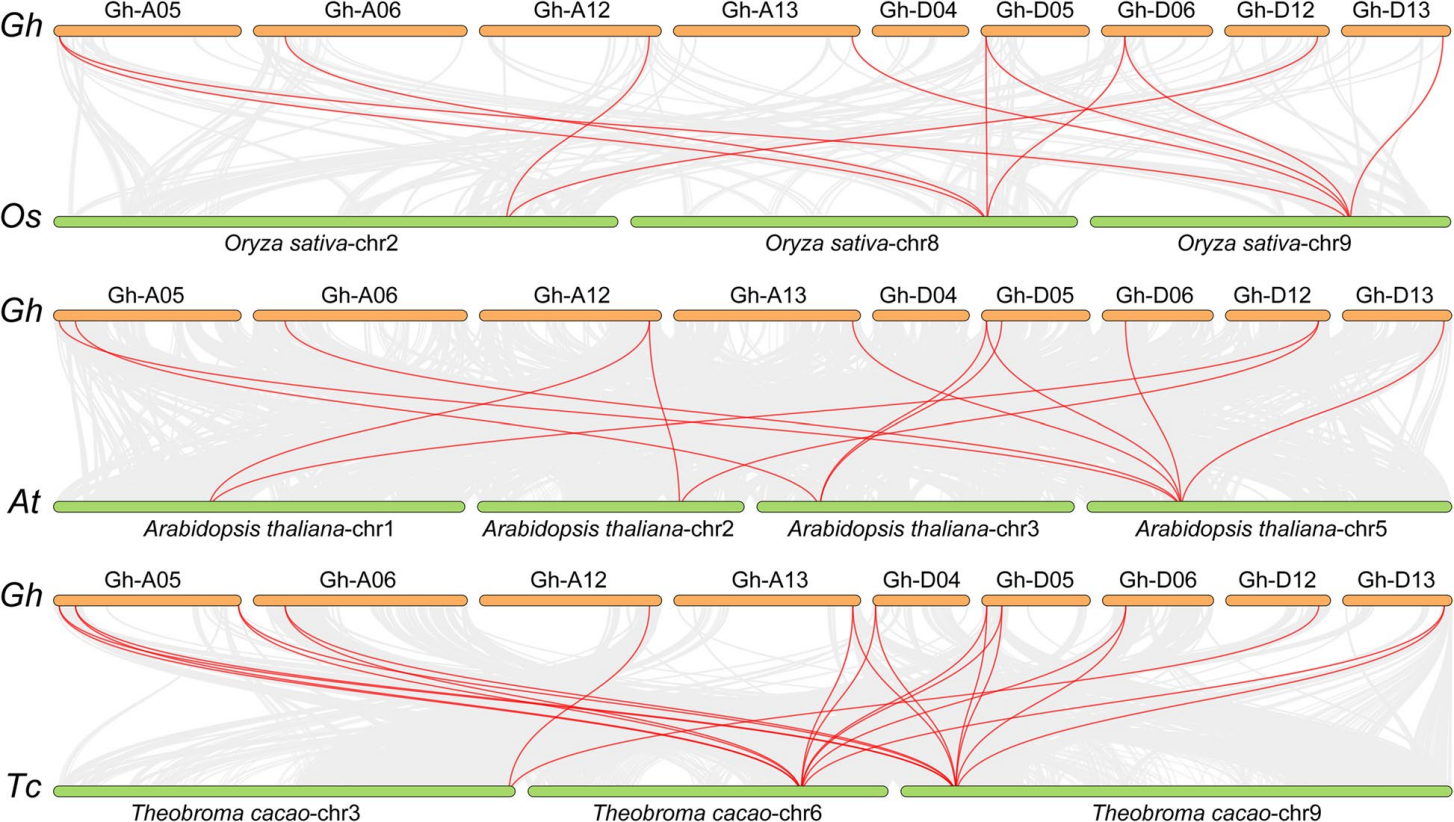
Syntenic analysis of *BRXL* gene between *G. hirsutum* and other three representative plant species. The gray line in the background represented the collinear blocks between *G. hirsutum* and three other plant species (rice, *Arabidopsis* and *Theobroma cacao*), while the red line exhibited the syntenic *BRXL* gene pairs. The species name with the prefixes of “*Gh*”, “*Os*”, “*At*” and “*Tc*”, indicate *G. hirsutum*, rice, *Arabidopsis thaliana* and *Theobroma cacao* respectively

Three comparative syntenic maps of *G. hirsutum* associated with three representative plant species, including one monocotyledonous plant (rice) and two dicotyledonous (*Arabidopsis* and *Theobroma cacao*), were constructed to further infer the phylogenetic relationships of the *BRXL* gene family in plants (Fig. 3; Additional file 3: Table S3). The results showed that a total of 8, 10 and 12 *GhBRXL* genes showed syntenic relationship with those genes in rice, *Arabidopsis* and *Theobroma cacao*, respectively (Fig. 3; Additional file 3: Table S3). The numbers of orthologous pairs anchored in the highly conserved syntenic blocks between *G. hirsutum* and the other three plant species (rice, *Arabidopsis* and *Theobroma cacao*) were 11, 13 and 22, respectively (Fig. 3; Additional file 3: Table S3). The number of *BRXL* collinear gene pairs between *G. hirsutum* and rice was similar to those in between of *G. hirsutum* and *Arabidopsis*, while the number of *BRXL* collinear gene pairs between *G. hirsutum* and *Theobroma cacao* was doubled. Interestingly, there was no *BRXL* collinear gene pairs on chromosome A04 between *G. hirsutum* and rice, and *G. hirsutum* and *Arabidopsis*, while there were *BRXL* collinear gene pairs, such as *GhBRXL3D*-*Thecc.06G117900.1* and *GhBRXL3D*-*Thecc.09G081200.1* (Fig. 3; Additional file 3: Table S3), found in *G. hirsutum* and *Theobroma cacao*, and suggested that these genes may have the important function in the *BRXL* gene family during evolution.

### Structure analysis of cotton *BRXL* genes

**Fig. 4:**
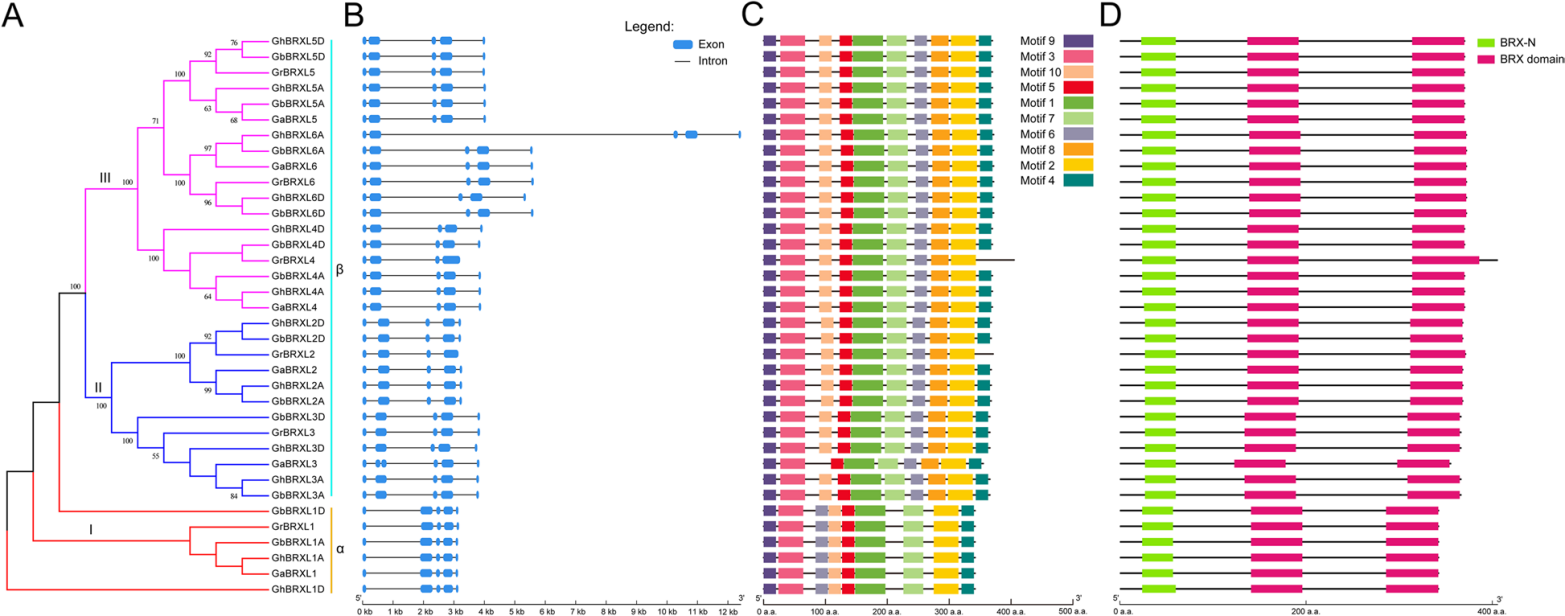
Gene structure, conserved protein motifs and domains analysis of BRXL proteins in four cotton species. **A** Phylogenetic tree constructed using MEGA 11.0 software based on four cotton species BRXL proteins. Details of subgroups were shown in different colors. **B** Exon-intron distribution of *BRXL* genes in four cotton species. Blue boxes indicate exons; black lines indicate introns. **C** Conserved motifs of proteins in four cotton species were identified with the MEME software. The 10 motifs were represented with different colored boxes. **D** Schematic representation of the conserved domains of BRXL proteins in four cotton species. The BRX-N domains and conserved BRX domains are highlighted by green boxes and red boxes, respectively. The length of DNA or protein sequence can be estimated using the scale at the bottom

The gene sequence structure, motif and protein amino acid structure of *BRXLs* in cotton were analyzed to predict their function (Fig. 4), and the results showed that all cotton *BRXL* genes in cotton contained 5 exons and 4 introns, except *GaBRXL3* (*GaBRXL3* contained 6 exons and 5 introns). Among them, the first intron of all *BRXLs* in α group was the longest in length, while the second intron in the DNA sequence of all *BRXLs* in β group was the longest in length (Fig. 4B). This result showed that genes belonging to the same phylogenetic branch exhibited similar gene structures, and different gene structures suggested different functions of *BRXLs*. A total of 10 motifs were set to screen the conserved domains in cotton BRXL proteins. Almost all BRXLs in each group showed the same or similar motifs, and 6 motifs (Motif 1, Motif 2, Motif 3, Motif 5, Motif 7 and Motif 9) were found to be the common motifs of all BRXL proteins in cotton (Fig. 4C, Additional file 8: Fig. S2), suggesting that these motifs were very conserved in BRXL. Similar to the *BRXL* gene structures, these motifs are divided into two different types based on the evolution of BRXLs. All BRXL proteins in the α group contained only 9 motifs (Motif 1, Motif 2, Motif 3, Motif 4, Motif 5, Motif 6, Motif 7, Motif 9 and Motif 10), without Motif 8 (Fig. 4C, Additional file 8: Fig. S2), whereas most of the BRXL proteins in the β group contained all 10 motifs, with the exception of GrBXL4, GrBRXL2, and GaBRXL3, which did not contain Motif 4, Motif 4 and Motif 10 respectively (Fig. 4C, Additional file 8: Fig. S2). These different motifs in BRXL suggested that the function of BRXLs might have diverged during evolution, even though these BRXL proteins all contained one BRX-N domain responsible for the subcellular localization of BRX proteins and two conserved BRX domains which are important to the regulatory activity of BRXL (Fig. 4D, Additional file 9: Fig. S3) [1–4].

### *Cis*-elements analysis of *GhBRXL* gene promoters

**Fig. 5:**
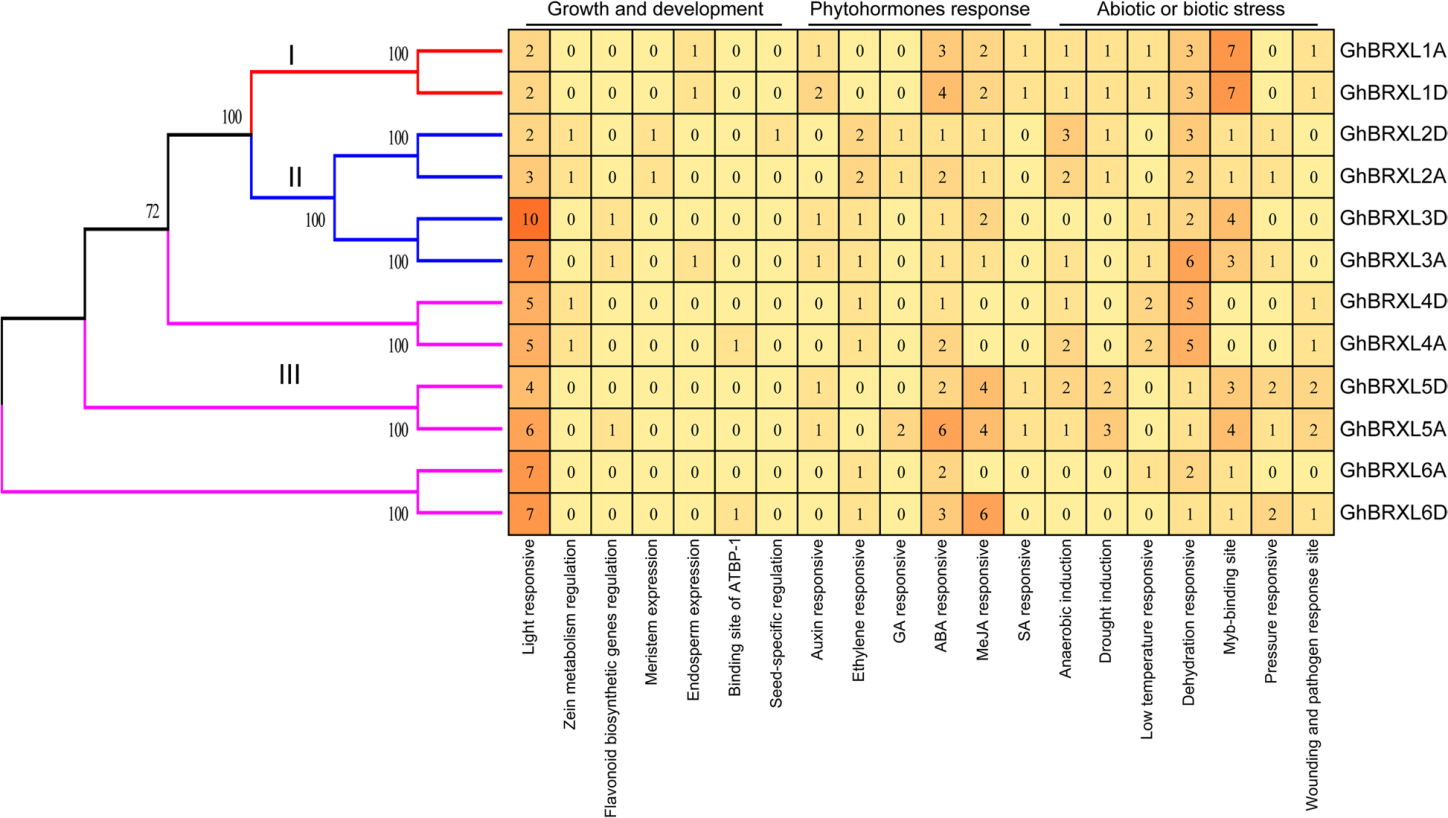
Analysis of *GhBRXL* genes promoter. Phylogenetic tree of GhBRXLs was generated using the MEGA 11.0. The number of *cis* -elements in promoters of *GhBRXLs* is represented by numerals in the boxes

The *cis*-elements within the 2000 bp sequences upstream of *GhBRXLs* were analyzed, and it was found that these *cis*-elements were mainly related to plant growth and development, phytohormones, stress response and so on (Fig. 5). Among the *cis*-elements in plant growth and development, most *GhBRXLs* contained light-related *cis*-elements, such as AT1-motif, etc., with the greatest number of light-responsive *cis*-elements in the promoter of the *GhBRXL3D* gene (Fig. 5). In addition, some specific *cis*-elements related to plant growth and development was found in *GhBRXLs*. For example, a seed-specific regulation element RY-element (*GhBRXL2D*), zein metabolism regulation related O_2_-site (*GhBRXL2A*, *GhBRXL2D*, *GhBRXL4A* and *GhBRXL4D*), flavonoid biosynthetic genes regulation related MBSI (*GhBRXL3A*, *GhBRXL3D* and *GhBRXL4D*), meristem expression related CAT-box (*GhBRXL2A* and *GhBRXL2D*), endosperm expression related GCN4_motif (*GhBRXL1A*, *GhBRXL1D* and *GhBRXL3A*), binding site of AT-rich DNA binding protein (ATBP-1) related AT-rich-element (*GhBRXL4A* and *GhBRXL6D*), and other cis-elements (Fig. 5). The phytohormone related *cis*-elements such as IAA (AuxR-core), ethylene (ERE), GA (GARE-motif), ABA (ABRE), MeJA (TGACG-motif) and SA (TCA-element), ABA and MeJA responsive *cis*-elements were the most enriched type of *cis*-element at the *GhBRXLs* promoter regions (Fig. 5). *GhBRXL5A* promoter had the highest number of ABA-responsive *cis*-elements, and *GhBRXL6D* promoter had the highest number of MeJA-responsive *cis*-elements. Dehydration responsive (MYC) and myb-binding site were predominant among biotic and abiotic stress related *cis*-elements at *GhBRXL* promoters (Fig. 5). In addition, there were *cis*-element of drought induction (MBS) (*GhBRXL5A* and *GhBRXL5D*) and low temperature responsive (LTR) cis-elements (*GhBRXL4A* and *GhBRXL4D*), which may be involved in regulating the genes expression in response to drought and temperature stresses. Other biotic and abiotic stress *cis*-elements, such as the anaerobic induction, pressure responsive member and wounding and pathogen response site were also found in this analysis. Above results suggested that *GhBRXL* genes might have diverged function in regulating cotton development, phytohormone response and stress response.

### Expression of *BRXL* genes in upland cotton

**Fig. 6:**
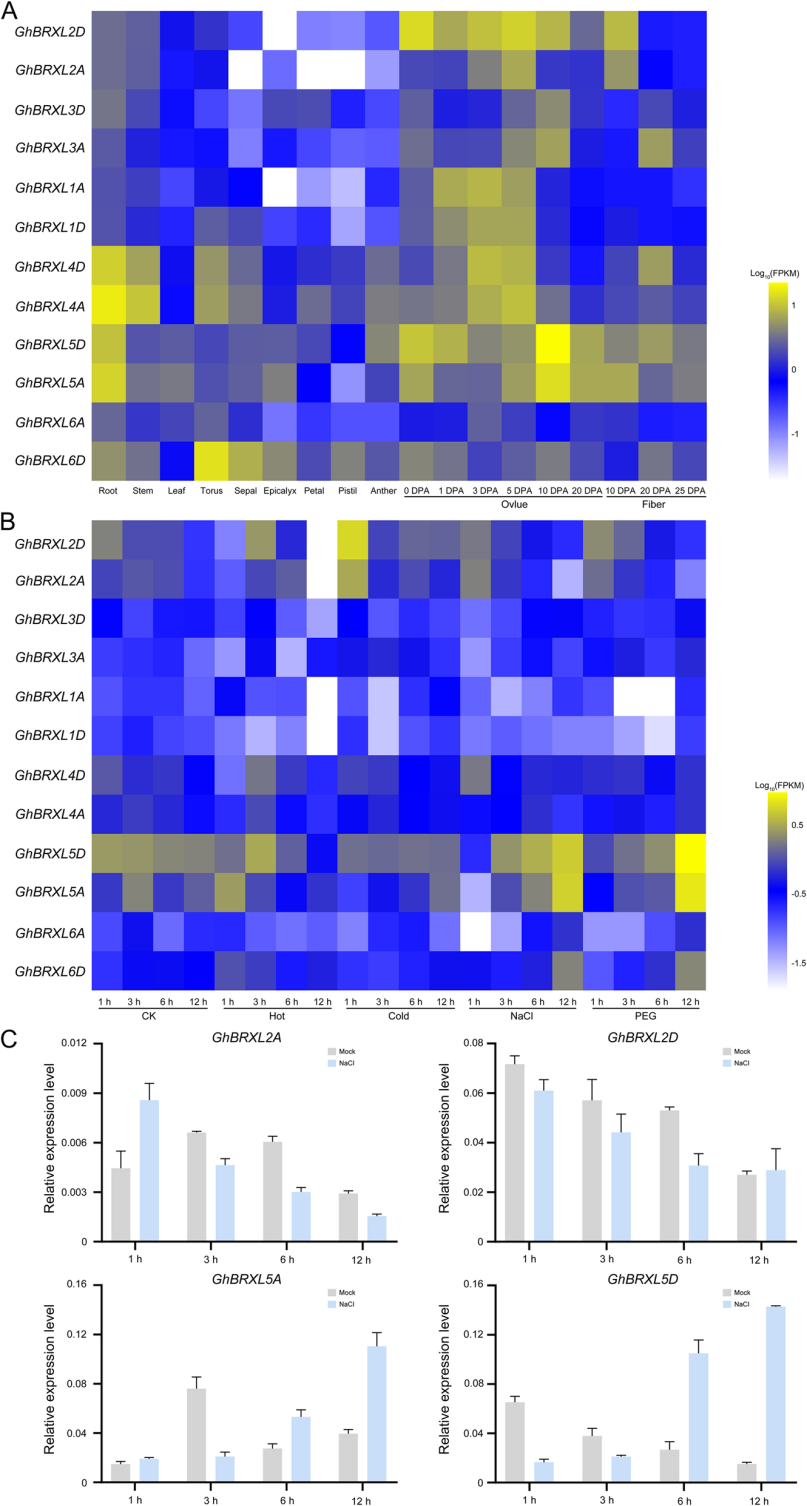
Expression level of *GhBRXLs* in different tissues and different stresses. **A** Expression profiles of *GhBRXL* genes in different tissues. **B** Differentially expressed *GhBRXL* genes under cold, hot, salt and PEG stress. CK represents the control cotton plants that were not treated with stress. The yellow in the figure represents high expression levels, and the blue indicates low expression levels. The heat map was generated based on logarithms of the FPKM values. **C** Relative expression of selected four *GhBRXL* genes under 200 mM NaCl stress was determined by qRT-PCR. The cotton *GhUBQ7* gene was used as internal control

We analyzed the transcriptome data of cotton from different tissues and found that some of the *GhBRXL* genes were mainly expressed in the ovule (Fig. 6A; Additional file 4: Table S4), for example, *GhBRXL1A*, *GhBRXL1D*, and *GhBRXL3D* were predominantly expressed in the ovule at different stage, and *GhBRXL1A* and *GhBRXL1D* could be used as candidate genes to study cotton ovule development and seed formation. *GhBRXL3A* was expressed not only in 5 DPA and 10 DPA ovules, but also in 20 DPA fiber (Fig. 6A; Additional file 4: Table S4). Similarly, *GhBRXL2A* and *GhBRXL2D* were expressed not only in ovule at different developmental stages, but also in cotton fibers elongation stage (10 DPA fibers) (Fig. 6A; Additional file 4: Table S4). *GhBRXL4A*, *GhBRXL4D*, *GhBRXL5A* and *GhBRXL5D* were expressed not only in ovule and fiber, but also in roots and stems (Fig. 6A; Additional file 4: Table S4). This suggested that these *GhBRXL* genes may have multiple functions involved in fiber development and plant architecture of regulation in addition regulating plant reproductive development.

Subsequently RNA-seq of *GhBRXLs* was analyzed under different stresses (hot, cold, NaCl and PEG) (Fig. 6B; Additional file 5: Table S5). Under hot stress treatment conditions, the highest expression level of *GhBRXL2D*, *GhBRXL4D* and *GhBRXL5D* showed at 3 h hot stress treatment, the highest expression level of *GhBRXL2A* showed at 6 h hot stress treatment, respectively, while the highest expression level of *GhBRXL5A* and *GhBRXL6D* showed at 1 h hot stress treatment (Fig. 6B; Additional file 5: Table S5). Under cold stress treatment conditions, the highest expression level of *GhBRXL2D*, *GhBRXL2A* and *GhBRXL4D* showed at 3 h cold stress treatment, while the highest expression level of *GhBRXL5A* showed at 12 h cold stress treatment (Fig. 6B; Additional file 5: Table S5). Compared with the control, the expression levels of *GhBRXL5D* all exhibited down-regulation under cold stress conditions (Fig. 6B; Additional file 5: Table S5). In NaCl and PEG treatment, *GhBRXL2A* and *GhBRXL2D* showed up-regulation at first and then down-regulated, with the highest expression at 1 h under stress treatment (Fig. 6B; Additional file 5: Table S5). On the contrary, the highest expression level of *GhBRXL5A*, *GhBRXL5D* and *GhBRXL6D* were observed at 12 h under stress treatment (Fig. 6B; Additional file 5: Table S5). In addition, *GhBRXL4D* only responded to salt stress treatment, with the highest expression at 1 h under NaCl stress treatment, and then decreased (Fig. 6B; Additional file 5: Table S5). Based on the above results, the expression levels of the different genes varied, indicated that the functions of different genes might be different. And the response of *GhBRXL* to different stress treatment implies that it has a potential molecular mechanism to adapt to adverse environmental conditions. Interestingly, we found that *GhBRXL2A*, *GhBRXL2D*, *GhBRXL5A* and *GhBRXL5D* responded in all four stresses (hot, cold, NaCl and PEG) (Fig. 6B; Additional file 5: Table S5), and *GhBRXL2A* and *GhBRXL2D* could be the candidate genes on the regulatory mechanism to temperature stress in cotton, and *GhBRXL5A* and *GhBRXL5D* could be the candidate genes to study the mechanism of salt and drought stress response in cotton. To further investigate the function of *BRXL* genes in response to salt stress, based on RNA-seq data, we selected four *GhBRXL* genes, *GhBRXL2A*, *GhBRXL2D*, *GhBRXL5A* and *GhBRXL5D* and designed specific primers for further qRT-PCR to verify of leaves from plants treated with NaCl the expression patterns of *BRXL* genes. The profiles of *GhBRXL* gene expression detected by qRT-PCR was showed to have similar trends with that analyzed using the RNA-seq data (Fig. 6B and C). This result suggested that they might involve in response to salt stress and their expression might be regulated by salt stress.

### Silencing of *GhBRXL5A* gene enhanced the resistance to salt stress

**Fig. 7:**
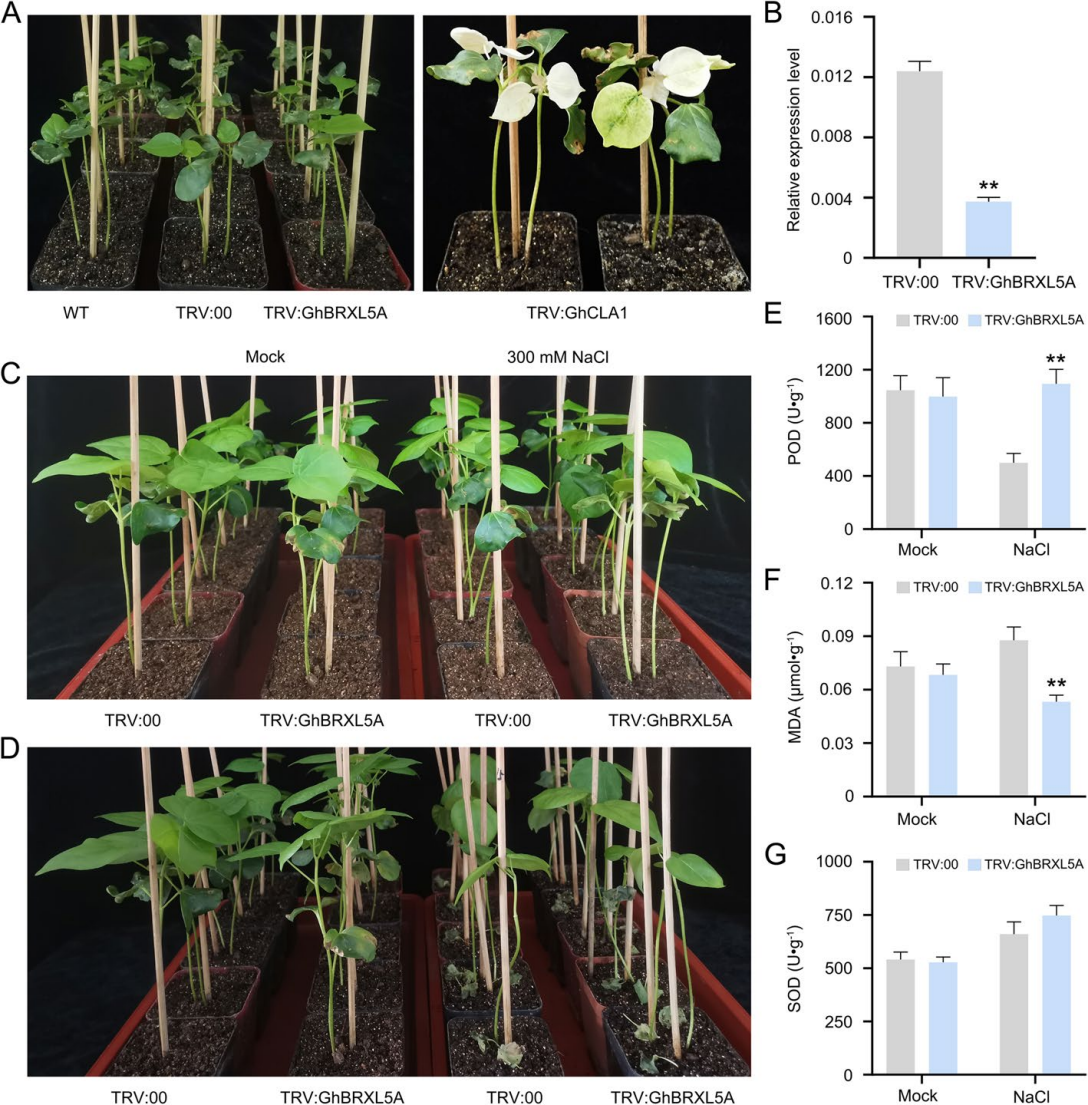
Silencing of *GhBRXL5A* improves the tolerance of cotton to salt stress. **A** The *GhBRXL5A* gene silenced, control (TRV:00) and positive control (TRV:*GhCLA1*) cotton plants before treatments. **B** Expression levels of *GhBRXL5A* in the silenced and control cotton plants. **C** and **D** Phenotype of cotton plants before and after treatments, **E** POD, **F** MDA contents and **G** SOD activity in the leaves of TRV:00 plants and TRV:GhBRXL5A plants under treatments. Asterisks indicate significant differences (independent t-tests): *, *P* < 0.05; **, *P* < 0.01

Based on the RNA-seq and qRT-PCR of *GhBRXL5A* gene, *GhBRXL5A* gene exhibited the highest expression level under 12 h of salt treatment (Fig. 6B and C), implying that the *GhBRXL5A* gene respond to salt stress. VIGS experiment was used to explore the function of *GhBRXL5A* in cotton under salt stress (Fig. 7). When the positive control plants (TRV:GhCLA) showed an albino phenotype (Fig. 7A), transcription of *GhBRXL5A* in the silenced plants (TRV:GhBRXL5A) was significantly reduced compared to the control (TRV:00) based on the qRT-PCR analysis (Fig. 7B), suggesting that *GhBRXL5A* gene was silenced in cotton. The control (TRV:00) and silenced plants (TRV:GhBRXL5A) were subsequently treated with the mock and salt stress (Fig. 7C-D), respectively. Phenotype of the silenced plants (TRV:GhBRXL5A) were not significantly different from that of the control plants (TRV:00) under the mock treatment, while fewer wilted and senescent leaves of silent plants (TRV:GhBRXL5A) were that of the control plants under salt stress (Fig. 7C-D). These results indicated that silencing of *GhBRXL5A* increased the tolerance of cotton plants to salt stress. To further investigate the response of *GhBRXL5A* to salt stress, we examined the changes of peroxidase (POD) and superoxide dismutase (SOD) enzyme activities, Malondialdehyde (MDA) content of *GhBRXL5A* silenced plants and the control plants under the mock and salt treatment (Fig. 7E-G), respectively. After the mock treatment, the contents of POD, SOD and MDA in the leaves of TRV:00 plants were no different from those of TRV:GhBRXL5A plants (Fig. 7E-G). After the salt stress treatment, POD enzyme activity in the leaves of TRV:00 plants were significantly lower than that of TRV:GhBRXL5A plants (Fig. 7E). On the contrary, MDA content in the leaves of TRV:00 plants were significantly higher than that of TRV:GhBRXL5A plants (Fig. 7F). Under salt stress treatment, there was no significant difference in SOD enzyme activity between the leaves of TRV:00 plants and TRV:GhBRXL5A plants (Fig. 7G), but SOD enzyme activity was increased in both TRV:00 plants and TRV:GhBRXL5A plants compared with the mock treated cotton plants. In conclusion, silencing of *GhBRXL5*A enhanced the tolerance of cotton to salt stress, suggesting that GhBRXL5A may be a negative regulator of salt stress.

### Association network

**Fig. 8:**
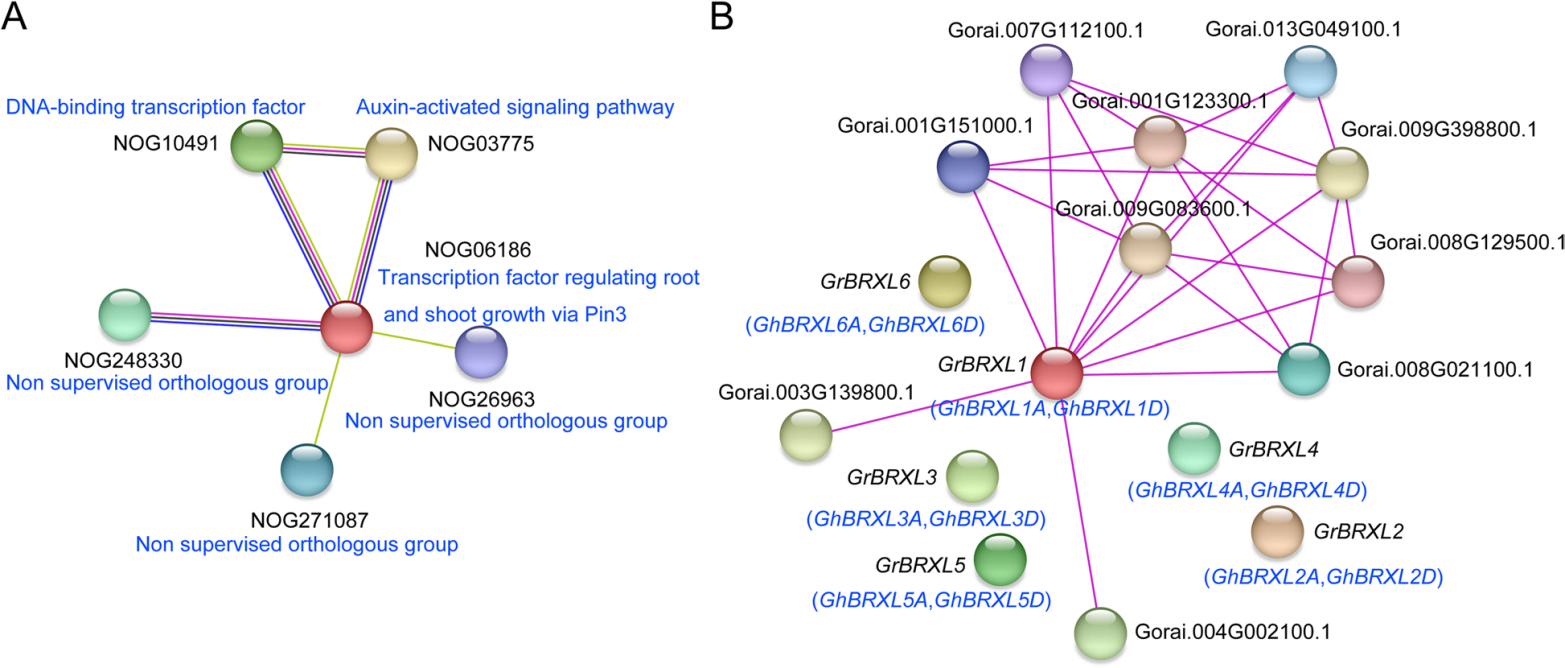
Association network of BRXL proteins. **A **Association network of BRXL proteins families. The blue letters represent BRXL proteins signaling pathway or other proteins families. **B** Association network of GrBRXL proteins with other proteins. The black and blue letters represent GrBRXL proteins and GhBRXL proteins, respectively

On the basis of the well-investigated *AtBRXs* in *Arabidopsis* [1–4], we analyzed and predicted the function of cotton BRXL proteins using STRING data (https://string-db.org/) (Fig. 8). In the protein families search, transcription factor involved in regulating root and shoot grown via PIN3 pathway (NOG06186) was found in the center (Fig. 8A), and other pathways related to the phenotypes, such as auxin-activated signaling pathway (NOG03775), suggested that the development of root and shoot involved in the regulation of auxin related signals. DNA-binding transcription factor activity pathway (NOG10491) was also found in the network, indicated that the BRXL protein may interact with other DNA-binding transcription factors to perform its function. In addition, there were some other pathways of unknown function, such as non-supervised orthologous group (NOG248330, NOG26963 and NOG271087).

In the single/multiple proteins sequences search, we found that the other GrBRXL proteins, except GrBRXL1 protein, did not form interaction networks with other proteins (Fig. 8B). The proteins that form a regulatory network with *GrBRXL1* proteins included Ethylene-responsive transcription factor RAP2-7 (Gorai.003G139800.1 and Gorai.004G002100.1), RING-H2 finger protein ATL47 (Gorai.008G021100.1 and Gorai.013G049100.1), CBL-interacting serine/threonine-protein kinase 9 (Gorai.001G151000.1, Gorai.007G112100.1 and Gorai.008G129500.1) and proteins of unknown function (Gorai.009G083600.1, Gorai.009G398800.1 and Gorai.001 G123300.1).

### Protein–protein interactions of GhBRXL

We selected four GhBRXL proteins, GhBRXL1A, GhBRXL1D, GhBRXL5A and GhBRXL5D, to perform yeast two-hybridization assays to analyze their potential homotypic and heterotypic interactions, in order to speculate whether these four *GhBRX* homologous genes were involved in related biological pathways or had overlapping functions. Based on amino acids sequence alignment between AtBRX and GhBRX proteins, the N-terminal truncated GhBRX proteins (GhBRXL1A^ΔN^, GhBRXL1D^ΔN^, GhBRXL5A^ΔN^ and GhBRXL5D^ΔN^) were cloned to the pGBKT7 vector as baits for interaction validation. The full-length proteins of respective genes were cloned to the pGADT7 vector used as preys.

**Fig. 9:**
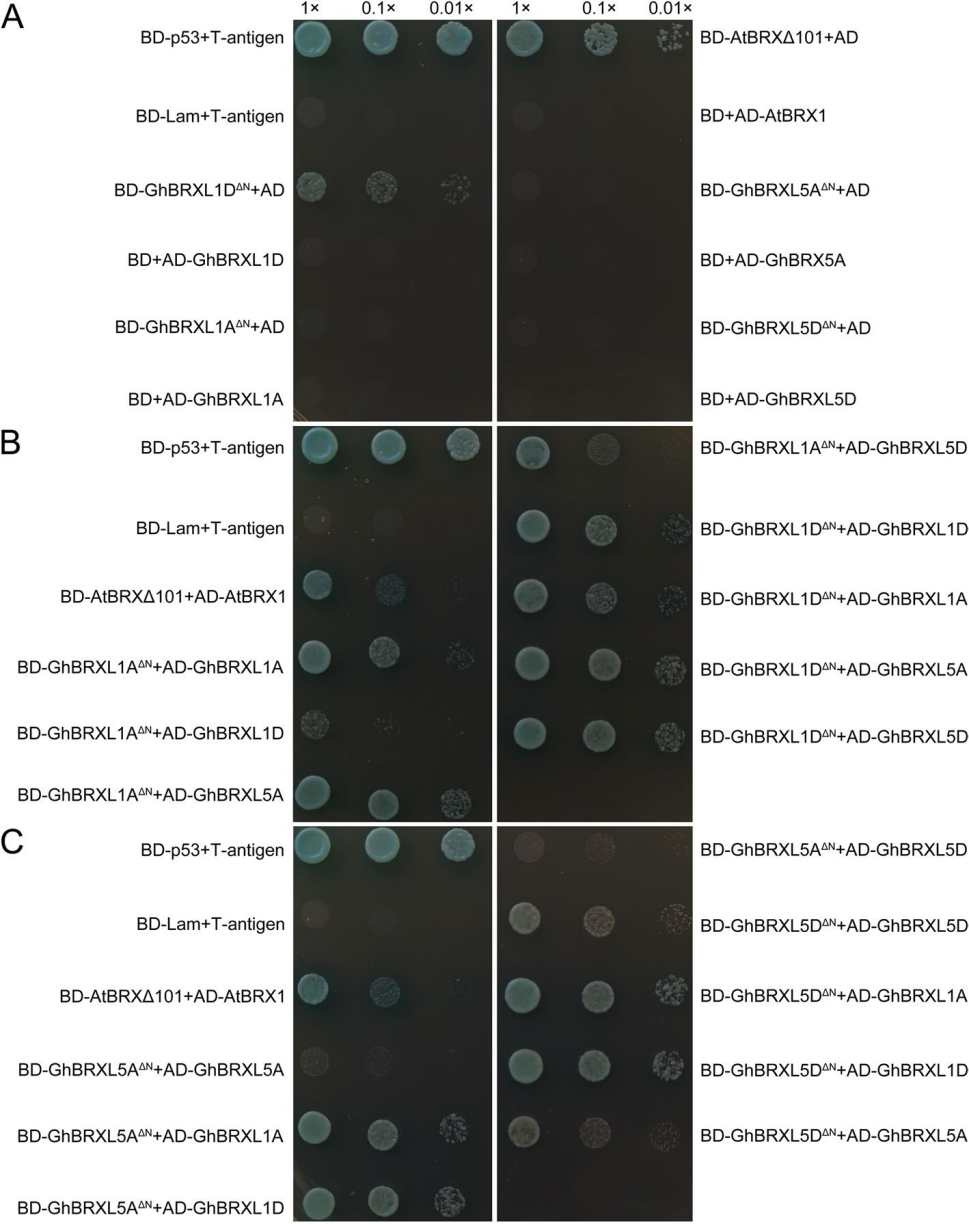
Homotypic and heterotypic interaction of BRX proteins. **A** BD-p53 and AD-T antigen as positive control and BD-Lam and AD-T-antigen as negative control, the bait (AtBRXΔ101, GhBRXL1A^ΔN^, GhBRXL1D^ΔN^, GhBRXL5A^ΔN^ and GhBRXL5D^ΔN^) and prey protein constructs (AD), the bait (BD) and prey protein constructs (AtBRX1, GhBRXL1A, GhBRXL1D, GhBRXL5A and GhBRXL5D) were co-transformed in yeast, respectively. Interactions were identified on SD-Trp-Leu-His-Ade (X-α-Gal and AbA). **B** The bait (GhBRXL1A^ΔN^ and GhBRXL1D^ΔN^) and prey protein constructs (AD-GhBRXL1A, AD-GhBRXL1D, AD-GhBRXL5A, AD-GhBRXL5D) were co-transformed in yeast, respectively. Interactions were identified on SD-Trp-Leu-His-Ade (X-α-Gal and AbA (1000 ng/mL)). Co-transformed BD:AtBRXΔ101 + AD:AtBRX1 was served as positive control. **C** The bait (GhBRXL5A^ΔN^ and GhBRXL5D^ΔN^) and prey protein constructs (AD-GhBRXL1A, AD-GhBRXL1D, AD-GhBRXL5A, AD-GhBRXL5D) were co-transformed in yeast, respectively. Interactions were identified on SD-Trp-Leu-His-Ade (X-α-Gal and AbA (1000 ng/mL)). Co-transformed BD:AtBRXΔ101 + AD:AtBRXL1 was served as positive control

The group (BD-AtBRXΔ101 and AD, BD-GhBRXL1D^ΔN^ and AD) could grow on the QDO (SD-Leu/-Trp/-Ade/-His/X-a-Gal/AbA) medium, while BD-p53 and AD-T antigen were the positive control, and BD-Lam and AD-T-antigen were the negative control. Meanwhile the other groups (BD-GhBRXL1A^ΔN^ and AD, BD and AD-GhBRXL1A, BD and AD-GhBRXL1D, BD-GhBRXL5A^ΔN^ and AD, BD and AD-GhBRXL5A, BD-GhBRXL5D^ΔN^ and AD, BD and AD-GhBRXL5D, BD and AD-AtBRX1) could not grow (Fig. 9A). These results indicated that the group (BD-AtBRXΔ101 and AD, BD-GhBRXL1D^ΔN^ and AD) have autoactivation, the other groups was not having autoactivation phenomenon. Therefore, to exclude autoactivation of BD-GhBRXL1D^ΔN^ and AD, the interaction was analyzed on QDO (SD-Leu/-Trp/-Ade/-His/X-a-Gal/AbA) medium containing higher concentration of AbA (1000 ng/mL). Since the interaction of AtBRXΔ101 with AtBRXL1 has been demonstrated [2], yeast co-transformed with BD-AtBRXΔ101 and AD-AtBRXL1 was used as positive controls, and for subsequent validation. Though there was an autoactivation with BD-GhBRXL1D^ΔN^ and AD, even at X-a-Gal/AbA yet heterotypic combination i.e., BD-GhBRXL1D^ΔN^ and AD-GhBRXL1A, BD-GhBRXL1D^ΔN^ and AD-GhBRXL5A, BD-GhBRXL1D^ΔN^ and AD-GhBRXL5D, showed more growth (Fig. 9B-C). This suggested higher affinity interaction of GhBRXL1D, with GhBRXL1A, GhBRXL5A and GhBRXL5D. Similarly, BD-GhBRXL1A^ΔN^ and AD-GhBRXL1A, BD-GhBRXL1A^ΔN^ and AD-GhBRXL5A, BD-GhBRXL1A^ΔN^ and AD-GhBRXL5D, BD-GhBRXL5A^ΔN^ and AD-GhBRXL1A, BD-GhBRXL5A^ΔN^ and AD-GhBRXL1D, BD-GhBRXL5D^ΔN^ and AD-GhBRXL5D, BD-GhBRXL5D^ΔN^ and AD-GhBRXL1A, BD-GhBRXL5D^ΔN^ and AD-GhBRXL1D, BD-GhBRXL5D^ΔN^ and AD-GhBRXL5A, also showed growth on the QDO (SD- Trp-Leu-His-Ade/X-a-Gal/AbA (1000 ng/mL)) medium (Fig. 9B-C). Yeast two hybridization results showed that higher affinity interaction of GhBRXL1A, with GhBRXL5A and GhBRXL5D; higher affinity interaction of GhBRXL5A, with GhBRXL1A and GhBRXL1D; higher affinity interaction of GhBRXL5D, with GhBRXL1A, GhBRXL1D and GhBRXL5A. In addition, homotypic (dimeric) interactions within GhBRXL1A and GhBRXL5D proteins were also observed yet these homotypic interactions.

## Discussion

The *BRX* family is a gene family with highly conserved plant-specific BRX domains. Previously, *BRX* family genes were have been identified and analyzed in many plant species, such as *Arabidopsis* [1–4], rice [5], wheat [11]. But the relevant information to understand the function of *BRX* genes is still limited. In this study, 81 *BRXL* genes from 12 plant species have been systematically identified and analyzed (Fig. 1; Additional file 1: Table S1), and these identified BRXL proteins vary in physicochemical properties such as MW, PI, and gene length. All BRXL proteins were predicted to be localized in the cell nucleus (Additional file 1: Table S1), indicating that they may function as the transcription factors in the nucleus [14]. And a total of 36 cotton *BRXLs* have been identified in four cotton species (Additional file 1: Table S1). Similar to the *BRX* gene family classification in other plant species [1–5, 9], these *BRXLs* were divided into two groups (α and β) (Fig. 1). *BRXL* genes on the same branch of the phylogenetic tree often have similar exon–intron structures, and *BRXL* members of the same subgroup exhibit conserved or similar motif distribution (Figs. 1 and 3), suggesting that cotton *BRXL* members of the same subgroup may have similar biological functions. Among different *BRXL* subgroups (Figs. 1 and 3), the gene structure and protein domain are different, which suggested that their functions may be different. The presence of these BRXL domains and motifs are consistent with the phylogenetic analysis, which corroborate the division of BRXL subgroups. Besides, the evolution of *BRXL* genes in cotton was dominated through the whole genome replication (Fig. 2; Additional file 2: Table S2), suggesting that each duplicate *BRXL* gene pair may have a closer evolutionary relationship and gene function.

The root system is an important organ to provide nutrient to support plant development. Some studies have shown that BRX mediates feedback between BR levels and auxin signaling in root growth [23]. *AtBRX* is involved in the cytokinin-mediated inhibition of lateral root initiation in *Arabidopsis* [3]. *Arabidopsis* mutant *brx-2* showed enhanced response to ABA mediated inhibition of root growth [13]. Auxin-responsive plasma membrane to nucleus movement of *BRX* suggests that *BRX* may provide a contextual readout to synchronize cellular growth with the auxin concentration gradient across the root tip [14]. As a cell proliferation and elongation regulator [1, 15], *BRX* is involved in the growth and development of plant roots through regulating the phytohormone level [3, 13, 14, 23]. In this study, analysis of *cis*-elements at the upstream promoter of *GhBRXL* has revealed that MeJA is one the major phytohormone predicted to induce *GhBRXL* expression, meanwhile ABA and auxin responsive *cis*-elements have also been identified, suggesting that the expression of *GhBRXL* genes were regulated by the induction of these phytohormone.

Cotton is a widely cultivated fiber and oil crop, however, the yield and quality of cotton are affected by abiotic adversities such as salt stress [24]. Therefore, improving the salt tolerance ability was one of the main directions of cotton breeding in the future, and the molecular mechanism of plant response to salt stress will provide important theory for breeding salt-tolerant cotton. *BRX* genes had been reported to be involved in the regulation of plant growth and development, as well as responses to biotic and abiotic stresses [3, 5, 6, 13, 14]. Studies have shown that rice *OsBRXL3* gene respond to drought and salt stress, whereas *OsBRXL2* and *OsBRXL5* gene only respond to cold stress [5]. *brx* mutants exhibit a salt-tolerant phenotype at a pH of 4.5 [6]. Here, through transcriptome data analysis, we discovered four *GhBRXL* genes (*GhBRXL2A*, *GhBRXL2D*, *GhBRXL5A* and *GhBRXL5D*) responsive to cold, heat, drought and salt stress (Fig. 6; Additional file 5: Table S5), suggesting that these four *GhBRXL* genes may be involved in the response to adversity stress, especially *GhBRXL5A* and *GhBRXL5D* genes are sensitive to salt stress. Interestingly, The ABA responsive element at the promoter region of GhBRXL5A and GhBRXL5D also suggests both genes may involve in salt stress response (Fig. 5). In addition, we have preliminarily verified the function of *GhBRX5A* gene through VIGS (Fig. 7), and have found that silencing *GhBRXL5A* gene enhance the resistance of cotton to salt stress. To investigate the possible mechanism of their response to salt stress, we analyzed some physiological parameters including accumulation of MDA and activity of POD, SOD both under salt stress and mock treatment (Fig. 7). The results of related biochemical parameters showed silencing of *GhBRXL5A* gene in cotton significantly affect the growth and physiology under salinity. MDA content was significantly decreased in *GhBRXL5A* silenced plants, whereas activity of POD activity was found to be increased significantly under salt stress. Under salt stress treatment, there was no significant difference in SOD activity between the control and *GhBRXL5A* silenced plants, but SOD activity was increased in both the control and *GhBRXL5A* silenced plants compared with the mock treated cotton plants. Abiotic stresses often result in overproduction of ROS, which interfering with normal physiological processes and ultimately leading to programmed cell death [25]. The higher POD activity and reduced MDA content after silencing of *GhBRXL5A* suggests silencing of *GhBRXL5A* gene regulated the scavenging ability of ROS, thereby enhancing plants tolerance to salt stress. Thus, these findings provided theoretical basis and genetic resources for breeding salt-resistant cotton genetic materials.

In addition, previous studies have reported that *BRX* genes involved in regulating plant architecture development, especially the plasticity of gravotropism, mainly through interacting with other membrane proteins and mediating the auxin signaling pathways [4, 7, 8, 10, 26]. Through the protein–protein interaction network, we have found that the cotton *BRXL* family may involve in regulating plant root and shoot development (Fig. 8), through pathways such as the PIN3 pathway (NOG06186) and the auxin-activated signaling pathway (NOG03775) [3, 15, 16]. Meanwhile, the GrBRXL interaction network revealed that GhBRLX1A and GhBRXL1D, the homologous of GrBRXL1 may interact with the ethylene-responsive transcription factor RAP2-7, the RING-H2 finger protein ATL47, the CBL-interacting serine/threonine-protein kinase 9, and so on (Fig. 8). Based on these results, we speculated that these *GhBRXL* genes may have the function of regulating cotton plant architecture.

Recently studies have shown that there are the homotypes and heterotypes of BRXL in *Arabidopsis* and wheat [1, 2, 11]. Here, we have also investigated the homotypic and heterotypic of GhBRXL proteins and have found that there were the homotypic and heterotypic interaction between GhBRXL proteins, such as GhBRXL1A and GhBRXL5D proteins (Fig. 9), speculated that the homotypic and heterotypic of BRXL protein may affect its function. These results provide useful information to understand the function of BRXL proteins.

## Conclusions

A total of 81 *BRXL* genes have been identified from 12 plant species, and the characteristics of *BRXL* gene family from four cotton species, including the analysis of the gene number, sequence structure and chromosomal location have been systematically investigated and analyzed. Most of cotton *BRXL* gene members have shared similar conserved structures of five exons and four introns. Most of cotton BRXL proteins have more than eight highly conserved motifs. All BRXL proteins contain one BRX-N domain and two BRX domains. Moreover, promoter cis-elements, and expression profiles of *GhBRXL* genes indicated that *GhBRXL2A/2D* and *GhBRXL5A/5D* were up/down-regulated in response to the different stress. *GhBRXL5A* was used as candidate gene to further verify their potential roles in salt stress response. Silencing of *GhBRXL5A* gene via VIGS improved salt tolerance in cotton plants, and have showed significantly changes in POD and MDA content levels, this suggests that the tolerance to salt stress was improved by regulating changes in POD and MDA content levels. Furthermore, yeast two hybrid analysis suggested homotypic and heterotypic interactions between GhBRXL1A and GhBRXL5D. Overall, these findings in this study provide insight into the potential functional roles of *BRXL* genes in cotton. The comprehensive analyses are helpful in selecting candidate *BRXL* genes for further functional characterization, and for the genetic improvement of the agronomic characters and environmental resistance in cotton.

## Material and methods

### Plant material and NaCl treatment

To detect the expression profile of *GhBRXL* genes by qRT-PCR, *G. hirsutum* cv. cotton cultivars 'TM-1' seedlings were grown in Hoagland solution under controlled conditions (28 °C,16 h light/8 h dark cycle) in the Cotton Research Institute of the Chinese Academy of Agricultural Sciences (China, Anyang). Cotton seedlings were treated with 200 mM NaCl at the 3-leaf stage, the true leaves were sampled at 1, 3, 6 and 12 h of the treatment and were immediately frozen in liquid nitrogen and stored for subsequent RNA extraction.

### Identification and characteristics of BRXL protein family

The reported *Arabidopsis* BRXL proteins and rice BRXL proteins were obtained from the *Arabidopsis* Information Resource (http://www.arabidopsis.org) and Phytozome (https://phytozome.jgi.doe.gov/pz/portal.html), respectively. Four cotton species (*Gossypium arboreum* (CRI), *G. raimondii* (JGI), *G. hirsutum* (ZJU) and *G. barbadense* (ZJU)) of BRXL proteins were acquired from the COTTONGEN (http://www.cottongen.org) [19], the Cotton Functional Genomics Database (https://cottonfgd.org/) [27] and the Cotton Omics Database (http://cotton.zju.edu.cn) [21] by blastp program using the amino acid sequence of these AtBRXL and OsBRXL proteins as query sequences. BRXL proteins from other species (*Zea mays*, *Sorghum bicolor*, *Vitis vinifera*, *Populus trichocarpa*, *Theobroma cacao* and *Glycine max*) were also retrieved using the above mentioned method from phytozome (https://phytozome.jgi.doe.gov/pz/portal.html). The BRX-N domain (IPR027988) and the BRX domain (IPR013591) of candidate BRXLs were further analyzed for using Interproscan 5 (http://www.ebi.ac.uk/interpro/) [28] and SMART (http://smart.embl.de/) [29].

Furthermore, the physicochemical characteristics (including molecular weight and isoelectric point) of these BRXL proteins were analyzed by the ExPASy tool (http://web.expasy.org/). The CELLO v2.5 server [30] was used to predict the subcellular localization site of each BRXL protein.

### Chromosomal distributions and synteny analysis of *BRXLs*

MapInspect software was used to map the chromosomes of *BRXL* gene localization in four cotton species [31]. The synteny and collinerity relationship between four cotton species genomes with default parameters were analyzed by the MCSCAN function in TBtools software [32]. The collinearity blocks across the whole genome were used to draw a collinearity map within BRXLs by the Dual synteny plot function of TBtools software [32].

### Sequences alignment and phylogenetictree construction

DNAMan 2.0 was used for multiple sequence alignment of amino acid sequences of AtBRXLs and cotton BRXLs. Phylogenetic trees of BRXL of 12 plant species were constructed using the neighbor-joining (NJ) method in MEGA 11.0 [33] with the default parameter settings. The same method was used to construct phylogenetic tree from conserved amino acid sequences of four cotton species and the model plants *Arabidopsis* and rice BRXs.

### Gene structure, conserved motifs and protein domains of BRXLs

The Multiple Em for Motif Elicitation (MEME) website (http://meme-suite.org/) was used to identify and analyze the conserved motifs of BRXL proteins with default parameters [34]. The MAST file generated from MEME website, the NWK file from phylogenetic tree analysis, the CDS and genome file from cotton BRXLs, were used as query, and the TBtools software [32] and the Gene Structure Display Server (GSDS) (http://gsds.cbi.pku.edu.cn/) [35] were used to draw the figure of phylogenetic tree along with the exon/intron structure, motifs and conserved domains of cotton BRXLs.

### Promoter regions and RNA-seq data of *GhBRXLs*

DNA sequences of 2000 bp upstream regions of *GhBRXLs* were obtained from CottonGen database (http://www.cottongen.org/) and defined as promotors [19]. *Cis*- elements in the *BRXL* genes promoter regions were predicted and analyzed through the PlantCARE website (http://bioinformatics.psb.ugent.be/webtools/plantcare/html/). RNA-seq data downloaded from the Cotton Omics Database (http://cotton.zju.edu.cn) were used to analyze differentially expressed genes under different tissues and stresses (Salt, PEG, Cold and heat) [21]. The heat map along with phylogenetic tree and *cis*-elements was generated through TBtool software by using fragments per kilo base of exon per million mapped fragments (FPKM).

### VIGS of *GhBRXL5A*

Cotton (*G. hirsutum* L.) cultivar ‘Zhongmiansuo 100’ was used in this study. Cotton plants were grown in the growth chambers (with a 16 h light/8 h dark cycle at 23℃) at Institute of Cotton Research of CAAS (Anyang, Henan, China). The 304 bp sequence fragments of *GhBRXL5A* were amplified from cDNA of *G. hirsutum* cv. Zhongmiansuo 100, and was digested with restriction enzymes *Bam*H I and *Sac* I, and subsequently introduced into the TRV:00 plasmid using the One Step Cloning Kit (Vazyme Biotech Co., Ltd, Nanjing, China) to generate the TRV: *GhBRXL5A* vectors. Primers of TRV:*GhBRXL5A* vector construction were shown in Additional file 6: Table S6. The TRV:*GhBRXL5A* and TRV:00 construct were inserted into *A. tumefaciens* strain GV3101 by electroporation. The primers used for vectors construction were listed in Additional file 6: Table S6. Followed by the protocol of VIGS experiment in cotton as mentioned previously by Shaban et al. [25], the constructs were injected to the cotton plants. When the *GhCLA1* gene silenced plants (positive control) displayed the albinism phenotype, which suggested that the target gene may have been silenced, then the VIGS efficiency was verified through qRT-PCR. After the positive control shows albino seedlings, 300 mM NaCl was applied to the root of the silenced plants and the control plants in the trefoil stage for 7 days, and the water treatment served as a mock. Three biological replicates were examined, with 20 plants in each replicate, and the results were analyzed using the student’s t-test.

### qRT-PCR

Based on the method described by RN38-EASYspin-Plus Plant RNA Kit (Aidlab Co., LTD, Beijing, China), total RNA of cotton leaves were extracted, and cDNA were generated using PrimeScript™ RT reagent Kit with gDNA Eraser (Takara Biomedical Technology Co., LTD, Beijing, China). The qRT-PCR specific primers for *GhBRXLs* and primers of internal reference *GhUBQ7* were shown in Additional file 6: Table S6. qRT-PCR were performed on the Bio-Rad 7500 fast fluorescence quantitative PCR platform with SYBR® Premix Ex Taq™ (Tli RNaseH Plus) (Takara Biomedical Technology Co., LTD, Beijing, China) in accordance with the manufacturer's protocol. Three independently biological replicates were set up, and each replicate in each experimental group contained 20 cotton plants. And 2^−ΔΔCt^ method [36] was used to measure relative expression levels of *GhBRXL* genes.

### Determination of biochemical parameters to salt stress

The MDA contents of 0.1 g cotton leaves from 300 mM NaCl treatment and mock treatment were determined according to the protocol described in the MDA measurement kit (BC0020, Beijing Solarbio Science & Technology Co., Ltd, Beijing, China). The activities of POD and SOD in cotton leaves were determined through the procedure described in POD (BC0090, Beijing Solarbio Science & Technology Co., Ltd, Beijing, China) and SOD measurement kits (BC0170, Beijing Solarbio Science & Technology Co., Ltd, Beijing, China), respectively. To determine the biochemical parameters, three biological replicates are used, and with 20 plants in each replicate, and the results were analyzed using the student’s t-test.

### Association network of the BRXL proteins

STRING software (https://string-db.org/) was used to analyze the interaction of BRXL proteins on the basis of the orthologs in *Arabidopsis* and *G. raimondii* with the confidence limit set at 0.4.

### Yeast two hybridization of GhBRXL proteins

To test protein–protein interactions in vitro, N-terminal truncated AtBRXΔ101 (343–1074 bp), GhBRX1A^ΔN^ (298–1026 bp), GhBRX1D^ΔN^ (298–1026 bp), GhBRX5A^ΔN^ (289–1110 bp) and GhBRX5D^ΔN^ (289–1110 bp) were cloned into pGBKT7 vectors (Clontech, CA, USA), while the full-length proteins of AtBRX1, GhBRX1A, GhBRX1D, GhBRX5A and GhBRX5D were cloned into pGADT7 vectors using gene-specific primers (Additional file 6: Table S6). The yeast two-hybrid assay was performed according to the manufacturer’s instructions (Shanghai Weidi Biotechnology Co., Ltd, Shanghai, China). Co-transformed *Saccharomyces cerevisiae* Y2HGold yeast cells were grown on SD/-T-L medium and incubated at 30 ℃ for 3d. Positive colonies were subsequently transferred to the selective and stringent SD/-L–T-H-A medium with X-a-Gal (20 mg/mL) and AbA (1000 ng/mL).

### Supplementary Information

Supplementary Material 1.

Supplementary Material 2.

## Data Availability

The datasets generated and/or analyzed in this work can be found in the COTTONGEN (http://www.cottongen.org), the Cotton Functional Genomics Database (https://cottonfgd.org/) and Cotton Omics Database (http://cotton.zju.edu.cn). The source data underlying the graphs in the main figures are available in supplementary information.

## Abbreviations

BRX: BRVIS RADIX
BRXL: BRX-like
BR: Brassinosteroid
ABA: Abscisic acid
*Gh*: *Gossypium hirsutum*
*Gb*: *Gossypium barbadense*
*Ga*: *Gossypium arboreum*
*Gr*: *Gossypium raimondii*
*At*: *Arabidopsis thaliana*
*Pt*: *Populus trichocarpa*
*Tc*: *Theobroma cacao*
*Vv*: *Vitis vinifera*
*Gm*: *Glycine max*
*Zm*: *Zea mays*
*Sb*: *Sorghum bicolor*
PI: Isoelectric points
MW: Molecular weights
a.a: Amino acids
WGD: Whole genome duplication
LTR: Low temperature responsive
DPA: Day post anthesis
MDA: Malondialdehyde
POD: Peroxidase
SOD: Superoxide dismutase
qRT-PCR: Quantitative real-time polymerase chain reaction
VIGS: Virus-induced gene silencing
AbA: Aureobasidin
MCSCAN: Multiple collinearity scan
FPKM: Fragments per kilo base of exon per million mapped fragments
MEME: Multiple em for motif elicitation
GSDS: Gene structure display server
