# Supplementary material for: Comprehensive identification and systematical characterization of BRX gene family and the functional of GhBRXL5A in response to salt stress
Source: BMC Plant Biol. 2024 Jun 11;24:528. doi: 10.1186/s12870-024-05220-3 (PMC11165835; doi:10.1186/s12870-024-05220-3)
Supplement: Supplementary file 2 — Supplementary Material 2. [file 12870_2024_5220_MOESM2_ESM.docx]

**Supplementary information**


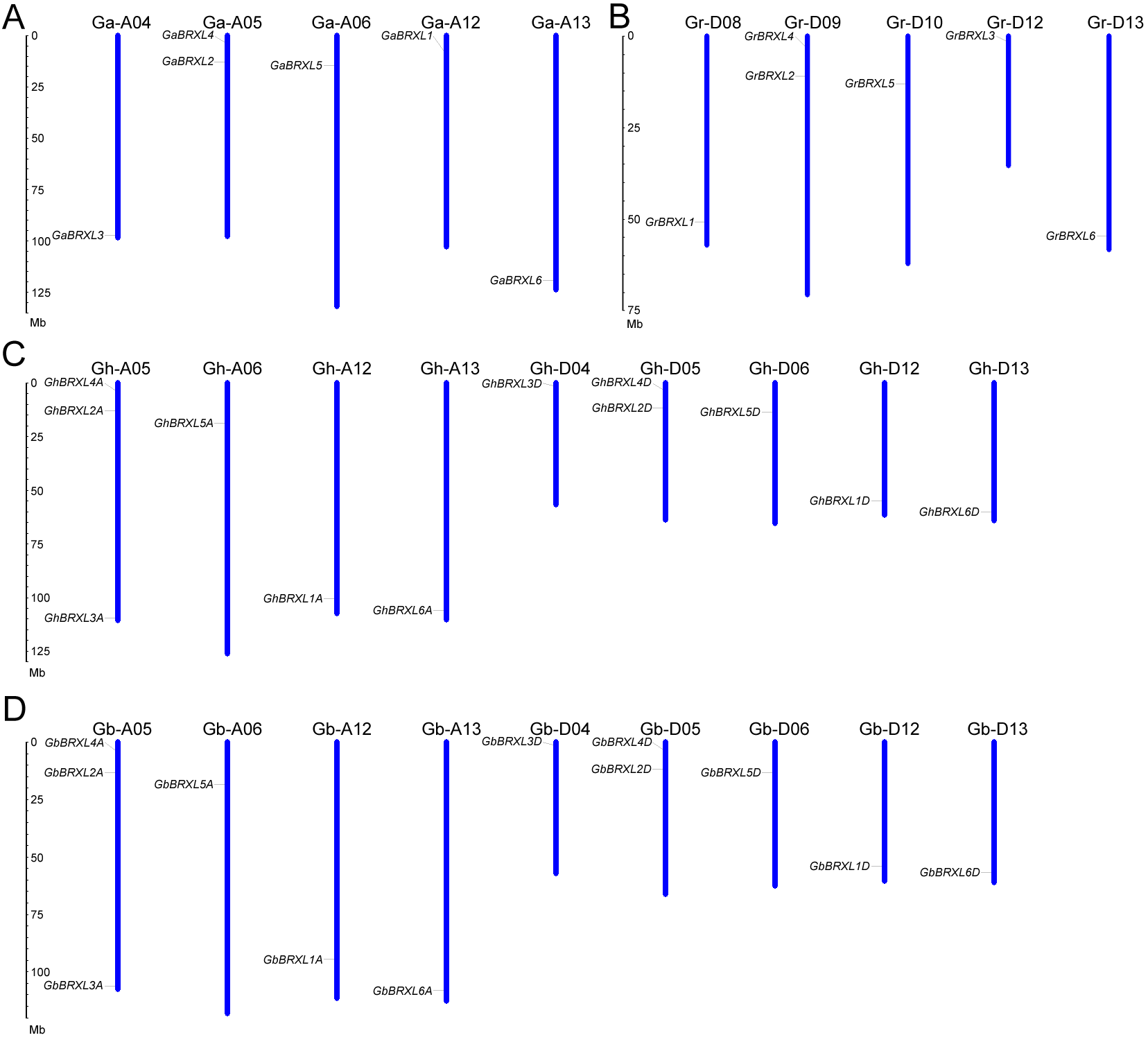


**Additional file 7: Fig. S1** Chromosomal localization of 36 *BRXL* genes in four cotton species.

(A) Chromosomal distribution of *GaBRXL*s on *G. arboretum*; (B) Chromosomal distribution of *GrBRXL*s on *G. raimondii*; (C) Chromosomal distribution of *GhBRXL*s on *G. hirsutum*; (D) Chromosomal distribution of *GbBRXL*s on *G. barbadense*.


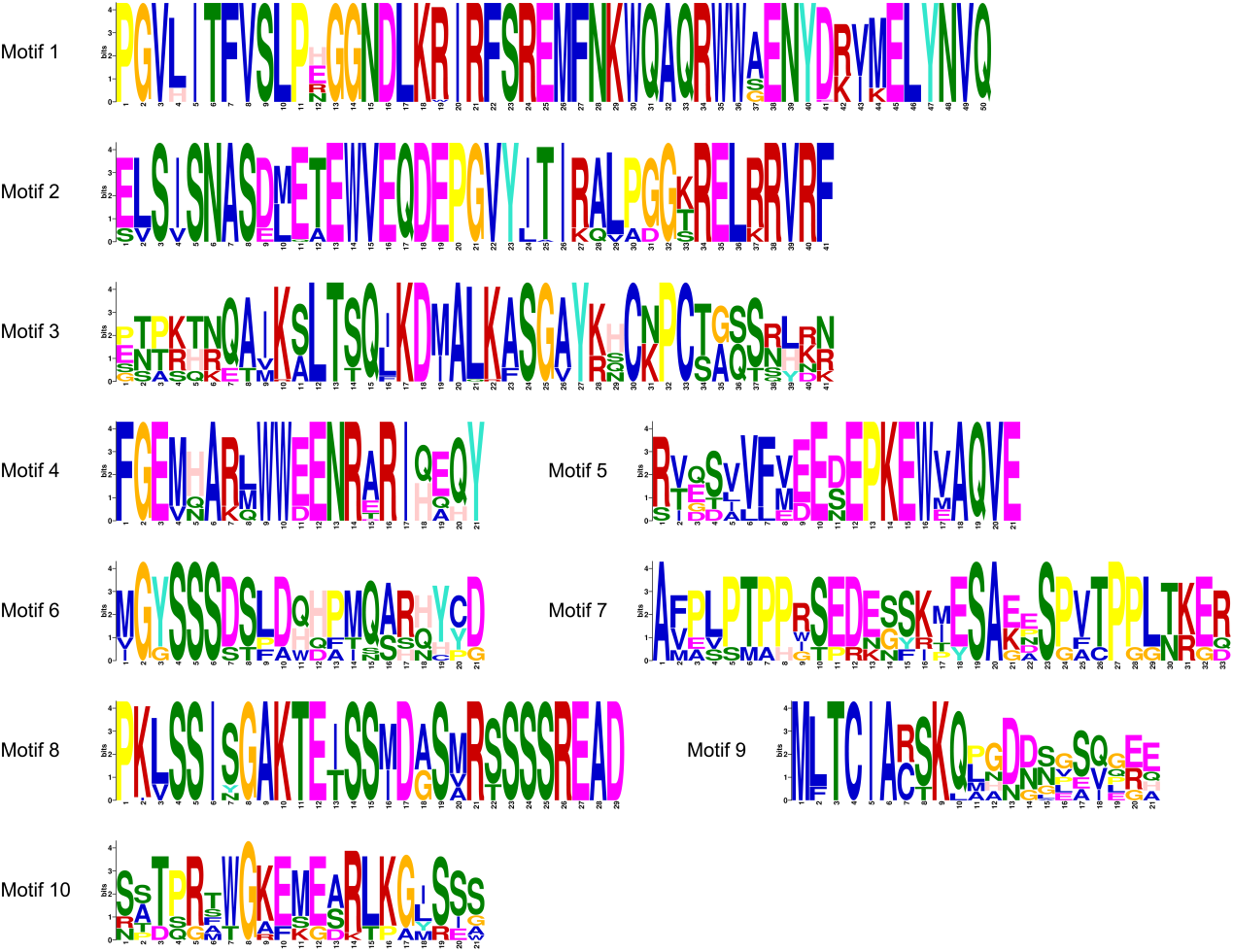


**Additional file 8: Fig. S2** Sequence logos of the 10 conserved motifs predicted from cotton BRXL proteins.

Different color letters represent different amino acid.


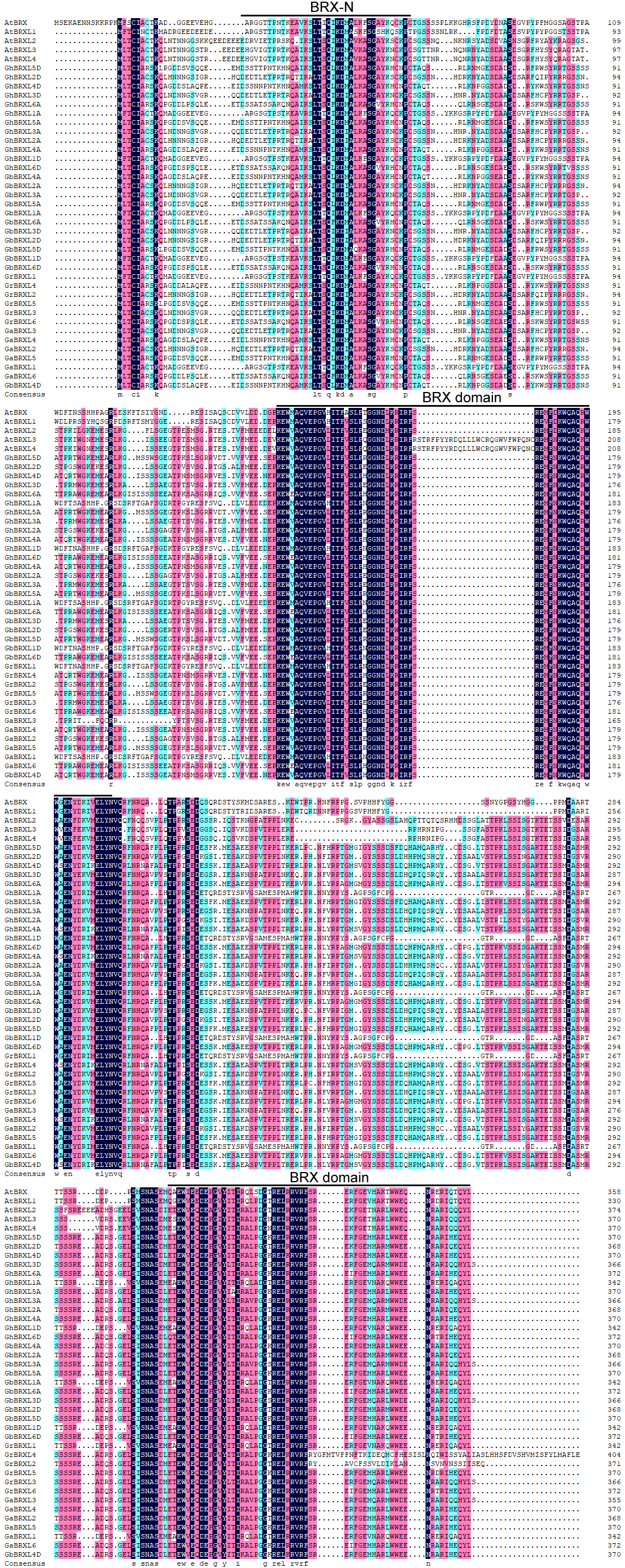


**Additional file 9: Fig. S3** Multiple sequence comparison of BRXLs protein between 4 cotton species and *Arabidopsis*.

Black line indicates one BRX-N domain and two BRX-domain.
